# Health Effect of Low-Dose-Rate Irradiation with Cumulative Threshold Dose: A Promising Area to Explore in Nuclear Emergency and Environmental Contamination

**DOI:** 10.3390/cells13181521

**Published:** 2024-09-11

**Authors:** Feng Ru Tang

**Affiliations:** Radiation Physiology Lab, Singapore Nuclear Research and Safety Initiative, National University of Singapore, Singapore 118415, Singapore; tangfr@gmail.com

## 1. Introduction

Humans live in an environment in which they are constantly exposed to meagre dose rates of radiation. Naturally high background radiation, nuclear accidents, leakage, weapon tests, terrorist attacks, or hospital stockpiles of nuclear waste may increase our environment’s radiation dose rate, leading either to increased chronic low- or high-dose-rate exposures with cumulative low- or high-dose radiation. The extrapolation of the adverse health effects of traditional acute high doses (≥100 mGy) with high-dose-rate irradiation (≥6 mGy/h), or of low doses with high-dose-rate radiation exposure, to low-dose-rate radiation with cumulative high or low doses may not be justifiable given that low-dose/dose-rate irradiation may induce hormesis and could stimulate protective biological responses. This includes the potential upregulation of DNA repair mechanisms and immune responses, which may prevent damage and reduce the baseline risk of certain diseases, including cancer. Fear of any dose of radiation exposure being harmful based on the linear no-threshold (LNT) paradigm has produced mass radiophobia, as is apparent in the public rejection of nuclear power plants as a means of producing clean energy; the forced evacuations of residents exposed to low-dose-rate radiation, leading to displacement-related stress, anxiety, and mortality; the refusal of necessary health check-ups using radiological imaging; and career discrimination. Therefore, research on the health effects of low-dose-rate radiation exposure with cumulative doses represents a crucial academic field with an important social and economic impact, irrespective of the difficulties faced in establishing a clear threshold due to the long latency periods of radiation-induced cancers and other non-cancerous diseases and the influence of environmental and genetic factors. Basing their decision-making on the actual effects of continuous low-dose-rate radiation exposure with cumulative doses, policymakers are empowered to make quick and scientifically grounded decisions as to whether mass evacuation is required once a nuclear or radiological emergency occurs, which may significantly reduce the unnecessary government costs flowing from the overestimation of the adverse health effects of accidental chronic low-dose-rate radiation exposure and the subsequent overaction.

## 2. Low-Dose-Rate Irradiation with Cumulative Doses

When considering radiation toxicity, radiation dose rate and cumulative dose are important, but their relative importance can depend on the context and specific biological endpoints being evaluated. The cumulative dose represents the total amount of radiation energy deposited in tissues over time. This is a critical factor in determining the overall risk of long-term effects such as cancer and genetic damage. For many chronic effects (for example, cancer), the cumulative dose is often used to estimate risk as it reflects the total exposure an individual has received. The relationship between cumulative dose and biological effects can be linear, threshold-based, or non-linear, depending on the specific type of radiation, radiation dose rate, and the biological response. Dose rate refers to the amount of radiation received per unit of time (e.g., mSv/hour). It is crucial in determining the immediate biological response to radiation. At lower dose rates, cells and tissues have more time to repair radiation-induced damage, potentially reducing the likelihood of harmful effects. High dose rates can overwhelm these repair mechanisms, leading to more significant damage. High dose rates over short periods of time (acute exposure) can cause immediate damage such as radiation burns, acute radiation syndrome, and severe cellular damage. Low-dose-rate irradiation over longer periods (chronic exposure) may allow for repair and adaptation, potentially leading to different biological outcomes. For acute radiation toxicity, such as radiation sickness, the dose rate is often more critical because the rapid delivery of radiation can cause immediate and severe biological damage; whereas for long-term effects like cancer, the cumulative dose tends to be the more relevant measure as it reflects the total exposure over time and the cumulative risk of developing radiation-induced health issues. Both the dose rate and the cumulative dose are important considerations, with dose rate influencing the effectiveness of biological repair mechanisms, being more critical for acute effects, and cumulative dose providing a measure of total exposure risk, being more relevant for chronic effects and long-term risk assessment. When either dose rate or cumulative dose is fixed or relatively consistent, it may constitute a threshold beyond which the other parameter may induce adverse health effects. The investigation of the threshold dose rate and accumulative dose may have significant scientific, social, and economic value. 

## 3. Linear No-Threshold (LNT)-Based Guidance May Do More Harm than Good in Reacting to Low-Dose-Rate Radiation Exposure after a Nuclear Emergency

Extensive radiation research evidence does not support the LNT paradigm, which asserts that any dose of radiation, no matter how small, carries some risk of causing harmful effects, with no safe threshold; whereas in fact, low dose/dose rate radiation may induce a beneficial effect and adaptive response in the human and animals [1,2,3,4,5,6,7]. Research on animals, such as mice, has shown that low doses of radiation can stimulate immune responses, enhance DNA repair mechanisms, and reduce tumor rates compared to non-irradiated controls [4,6]. Two years after the Chernobyl nuclear accident, the population of wild boars in the 30 km around nuclear power plant was eight times over the pre-accidental level, and the population of elk, deer, wolf, fox, and mouse-like rodents increased significantly by the spring of 1988 [8,9]. Geras’kin et al., (2008) reviewed the effect of non-human species irradiation after the Chernobyl nuclear power plant accident, indicating that the Chernobyl exclusion zone was a breeding area for white-tailed eagle, spotted eagle, eagle owl, crane, and black stork after the accident, suggesting the positive ecological consequences of the most severe nuclear accident in the history [10]. Low-dose-rate gamma-irradiation protects fruit fly chromosomes from double-strand breaks and telomere fusions [11] and promotes the growth of silkworms [12]. Epidemiological studies suggest lower cancer rates and longer lifespans in populations exposed to higher natural background radiation when compared to those in lower background areas [5,6,7]. Studies of nuclear workers have not consistently shown increased cancer risks at low doses, suggesting a possible threshold effect. Data from Hiroshima and Nagasaki show increased cancer risks primarily at high doses, with some uncertainty about the effects at low doses. Areas with high natural background radiation, such as Ramsar, Iran, do not show significantly higher cancer rates than areas with lower radiation levels. These data potentially indicate a threshold or hormetic response. Furthermore, some original research data from those LNT advocators could not support the LNT hypothesis [13,14,15,16,17]. While the call for ending the LNT model, the greatest scientific scandal of the 20th century [18], has been made for many years [13,14,19,20,21,22,23,24,25,26], the public still associates the word radiation with harm and has poor acceptance of the radiation threshold, despite there being no convincing scientific evidence to indicate thresholds after different low-dose-rate radiation exposures [27]. To reduce public anxiety and adverse social effects, policymakers may have to take extra precautions and overreact to accidents which may cause unnecessary costs and social burdens. Improper preparation for evacuation may also cause high mortality in individuals with severe medical problems, as well as neuropsychiatric disorders and abortion. Given that the public does not have high levels of knowledge of low-dose ionizing radiation, it is time to focus radiation research on low-dose-rate exposure with cumulative doses to determine the relevant thresholds so that scientific research data can educate the public. Policymakers may rely on these data to make quick and scientifically grounded decisions without fomenting distrust the public.

After the Fukushima nuclear accident, no mortality was caused by radiation exposure per se in the exposed populations. However, there has been increased mortality among displaced elderly people with other serious health problems during the evacuation. The evacuated population also suffered from mental health, lifestyle-related health problems, and social issues after the accident. In the first few days after the Fukushima accident, the evacuation of patients with severe medical diseases, such as end-stage renal failure or stroke, from a 20 km radius around the damaged Fukushima Daiichi nuclear power plant caused the death of more than 50 patients either during or soon after evacuation, mainly due to hypothermia, dehydration, and the deterioration of underlying medical problems, but not the radiation itself. Ill-prepared evacuation might increase the health risk of hospital inpatients or elderly people [28]. The difference between mortality rates in the Fukushima regions in 2010 and 2011 was exceptionally high during the first 3 months. The mortality rate was 2.4 times higher in 2011 than in 2010 [29]. More than a thousand people died from causes related to the evacuation and the continued exclusion of residents from their homes for extended periods. This occurred even though no significant contamination was found in the patients evacuated [30]. Given the fact that the elderly are more radioresistant than the younger generation, and that low dose irradiation may treat various disorders, such as Alzheimer’s disease (AD) and Parkinson’s disease (PD) [31,32,33], leaving those displaced elderly people with serious health problems in their original home or hospital with low-dose-rate radiation exposure may be more beneficial than harmful as it may improve their severe chronic disorders.

## 4. Low-Dose-Rate Radiation Exposure with Cumulative Threshold Doses: A Promising Area to Explore

The current dose limits for different organs or tissues are based on the threshold doses for morbidity in specific organ systems and for mortality. These threshold doses are derived from past events and experiences, generally at high dose ranges with high-dose-rate radiation exposure [34]. The analysis of the leukemia incidence data of Hiroshima survivors identified the acute dose threshold at about 0.7 Sv or 0.7 Gy [35]. In dogs exposed lifelong to cobalt-60 r-radiation, the dose rate threshold for life span reduction is about 600 mGy per year, ranging from 300 to 1100 mGy per year [36]. Early studies showed that chronic or fractionated low-dose-rate irradiation with high cumulative dose prolonged the lifespan [37,38,39,40,41,42], though this finding was not supported by other research teams. For instance, Tanaka’s group reported that the lifespans of mice of both sexes irradiated with 21 mGy/day and females irradiated with 1.1 mGy/day for 400 days were significantly shorter than those of the control group [43,44], suggesting the existence of threshold doses between hormetic and adverse health effects. Similarly, chronic in utero exposure to gamma rays at dose rates below 20 mGy/d for the entire gestation period does not induce obvious harmful health effects at both the early- and late-adult-life stages of B6C3F1 mice [45,46]. However, the induced histopathological changes at different life stages of the animal were observed after prenatal exposure to medium dose rates of 200 mGy/d and 400 mGy/d and to total accumulated doses of 3600 mGy and 7200 mGy [26], respectively, suggesting that a threshold dose rate/cumulative dose may exist from 20 to 200 mGy/d or from 360 to 3600 mGy when one of the parameters is fixed. Therefore, investigation of the health effects of low-dose-rate irradiation with cumulative threshold doses should prove a promising area to explore within broader domain of nuclear emergency and environmental contamination, with significant scientific, social, and economic value. 
